# Regulation Efficacy and Mechanism of the Toxicity of Microcystin-LR Targeting Protein Phosphatase 1 via the Biodegradation Pathway

**DOI:** 10.3390/toxins12120790

**Published:** 2020-12-11

**Authors:** Luyao Ren, Zhengxin Hu, Qian Wang, Yonggang Du, Wansong Zong

**Affiliations:** College of Geography and Environment, Shandong Normal University, 88# East Wenhua Road, Jinan 250014, China; aaarlyao@163.com (L.R.); h17862181566@163.com (Z.H.); wqsdnu@163.com (Q.W.); zhiyuan_du2009@163.com (Y.D.)

**Keywords:** microcystins, biodegradation products, toxicity regulation, protein phosphatase 1

## Abstract

Biodegradation is important to regulate the toxicity and environmental risk of microcystins (MCs). To explore their regulation effectiveness and mechanism, typical biodegradation products originating from microcystin-LR (MCLR) were prepared and purified. The protein phosphatase 1 (PP1) inhibition experiment showed the biodegradation pathway was effective in regulating the toxicity of the biodegradation products by extending the biodegradation. With the assistance of molecular docking, the specific interaction between the toxins and PP1 was explored. The MCLR/MCLR biodegradation products combined with PP1 mainly by the aid of interactions related to the active sites Adda^5^, Glu^6^, Mdha^7^, and the ionic bonds/hydrogen bonds between the integral toxin and PP1. As a consequence, the interactions between Mn_2_^2+^ and Asp_64_/Asp_92_ in the catalytic center were inhibited to varying degrees, resulting in the reduced toxicity of the biodegradation products. During the biodegradation process, the relevant key interactions might be weakened or even disappear, and thus the toxicity was regulated. It is worth noting that the secondary pollution of the partial products (especially for Adda^5^-Glu^6^-Mdha^7^-Ala^1^ and the linearized MCLR), which still possessed the major active sites, is of deep concern.

## 1. Introduction

Microcystins (MCs), produced by cyanobacteria, pose a significant threat to public health and raise concerns about the safety of drinking water [1,2]. MCs are a class of cyclic heptapeptides that share a general structure of cyclo-D-Ala^1^-X^2^-D-isoAsp^3^-Z^4^-Adda^5^-D-isoGlu^6^-Mdha^7^ [3]. X^2^ and Z^4^ are two variable L-amino acids, Adda^5^ is (2S, 3S, 8S, 9S)-3-amino-9-methoxy-2,6,8-trimethyl-10-phenyldeca-4(E), 6(E)-dienoic acid, and Mdha^7^ is N-methyl-dehydroalanine. Among the variants of MCs, MCLR (L and R stand for Leu^2^ and Arg^4^, respectively) is the most toxic and has been widely used as the standard variant [4,5].

MCs tend to be absorbed by hepatic cells and induce acute liver damage through potent inhibition of protein phosphatase 1 and 2A (PP1 and PP2A, major regulators of protein dephosphorylation) [6,7]. MCs undergo a two-step interaction with PP1/PP2A: the first step involves a reversible binding of MCs to the hydrophobic cage adjacent to the active site pocket; the second step involves the formation of covalent bonds between the Mdha^7^ residue and the nucleophilic sites (cysteine residues), leading to the irreversible inactivation [8,9]. The inhibition of PP1/PP2A leads to the accumulation of phosphorylated proteins in hepatic cells, causing cell necrosis, massive hemorrhage, and death [3,5,10].

Due to the hepatotoxicity of MCs, controlling their levels is of great importance. Compared with conventional water treatment methods, biodegradation was the first barrier for MC pollution and thus deserve great attention. Fundamental knowledge and application development of MC biodegradation have been widely reported, including for the natural organisms and species involved, its molecular mechanisms, and application potential [11]. MCs can be degraded by dozens of bacterial strains from natural water bodies and sediments, with the majority identified as *Sphingomonas* spp., *Sphingopyxis* spp., *Novosphingobium* spp., and *Bacillus* spp. [12]. With the characterization of the gene cluster encoding MC biodegradation, four genes were sequentially identified, namely, mlrC, mlrA, mlrD, and mlrB [13]. The gene mlrA respond to the cleavage of the peptide bond Z^4^-Adda^5^, forming linearized MCs. Genes mlrB and mlrC respond to the sequential cleavage of the peptide bonds Ala^1^-X^2^ and Adda^5^-isoGlu^6^, respectively. Relevant degradation products include Adda^5^, hexapeptide, tetrapeptides, tripeptides, and so on [14]. The gene mlrD respond to the active transport of MCs and its degradation products [11]. In this way, the ring structure of the MCs is destroyed and the first interaction step between the MCs and PP1/PP2A might be blocked.

Though biodegradation could regulate the toxicity of MCs, information about the residual toxicity of the biodegradation products, the structure–toxicity relationships of the biodegradation products, and the detoxification mechanism associated with PP1 and PP2A is relatively limited. To regulate the potential threat of MCs in a comprehensive way, clarifying the detoxification effectiveness and molecular mechanism via the biodegradation pathway is of great importance.

To fill the research gap in this field, several typical biodegradation products originating from MCLR were isolated and identified by MS (mass spectrometry) and MS/MS (tandem mass spectrometry) analyses. After chromatography preparation and purification, the biological toxicity of the biodegradation products of MCLR was evaluated by a PP1 inhibition assay. On the basis of molecular simulation, the key active sites and modes for the interaction between the MCLR/MCLR biodegradation products and PP1 were identified. Taking the biological toxicity and interaction models into consideration, the regulation mechanism of the MC biodegradation pathway was explored.

## 2. Results and Discussion

### 2.1. MCLR Biodegradation Products Identification

**Figure 1:**
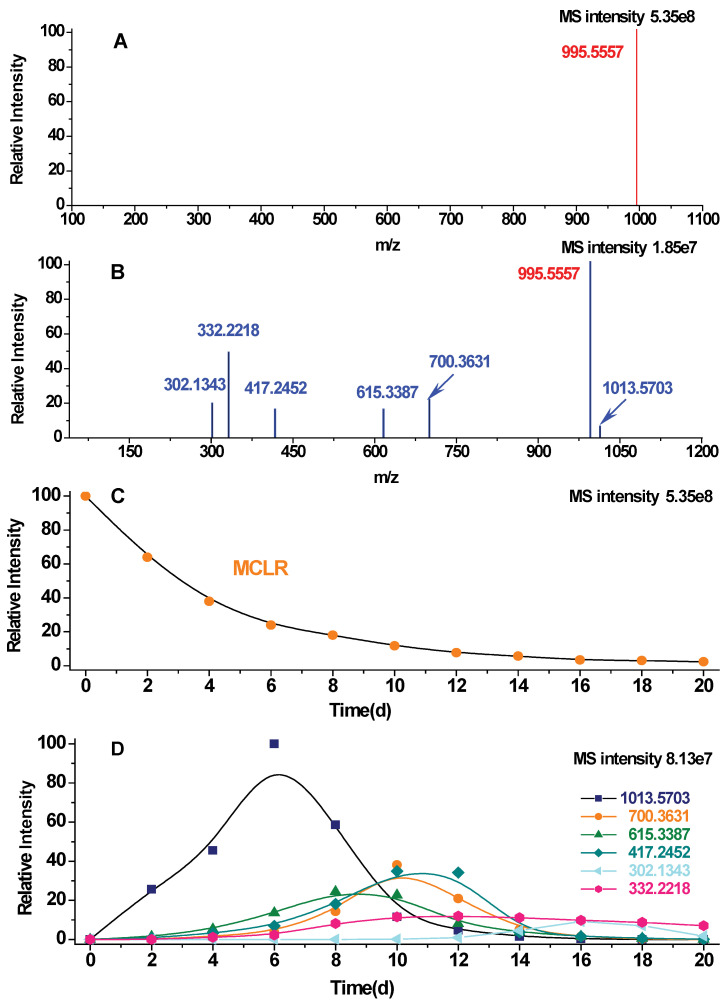
MS detection of MCLR and MCLR biodegradation products: MS spectrum for the crude extract of *M. aeruginosa* FACHB-905 (**A**) and the biodegradation sample (for 14 days) (**B**); MS intensity changes for the MCLR (**C**); and typical MCLR biodegradation products (**D**) (the fitting curve are also plotted).

The biodegradation of MCLR was related to the cleavage of peptide bonds, Ala^1^-Leu^2^, Arg^4^-Adda^5^, and Adda^5^-isoGlu^6^ [15]. As the theoretical molecular weights of the structural units, -Ala-, -Leu-, -MeAsp-, -Arg-, -Adda-, -isoGlu-, and -Mdha-, are 71.0365 Da, 113.0835 Da, 129.0420 Da, 156.1005 Da, 313.2036 Da, 129.0420 Da, and 83.0365 Da, the *m*/*z* signals for the single-protonated MCLR biodegradation products, linearized MCLR (Adda^5^-Glu^6^-Mdha^7^-Ala^1^-Leu^2^-MeAsp^3^-Arg^4^), isoGlu^6^-Mdha^7^-Ala^1^-Leu^2^-MeAsp^3^- Arg^4^, Adda^5^-isoGlu^6^-Mdha^7^-Ala^1^, Leu^2^-MeAsp^3^-Arg^4^, isoGlu^6^-Mdha^7^-Ala^1^, Adda^5^, should be 1013.5709, 700.3634, 615.3391, 417.2457, 302.1347, and 332.2220, respectively. To avoid the interference of impurities, a selected ion scan model was used to identify the above biodegradation products with an ultra-high-resolution mass spectrometer (relative mass error <2 ppm). Figure 1A shows that MCLR (*m*/*z* = 995.5557) was the only detected ion for the crude extract of *M. aeruginosa* FACHB-905. However, in the MS spectra of the biodegradation samples, the MCLR biodegradation products were continuously detected (Figure 1B–D). By extracting the intensity of the MS signals, the changed MS intensity of the MCLR and above biodegradation products also could be obtained. With the extension of the biodegradation process, the MCLR biodegradation products exhibited peak values at different times, while MCLR gradually decreased (Figure 1D). According to the peak times, the linearized MCLR should be the original degradation product of MCLR. Adda^5^, isoGlu^6^-Mdha^7^-Ala^1^-Leu^2^-MeAsp^3^-Arg^4^, Adda^5^-isoGlu^6^-Mdha^7^-Ala^1^, and Leu^2^-MeAsp^3^-Arg^4^ should be the secondary degradation products of linearized MCLR. Adda^5^ and IsoGlu^6^-Mdha^7^-Ala^1^ should be the secondary degradation products of Adda^5^-isoGlu^6^-Mdha^7^-Ala^1^.

**Figure 2:**
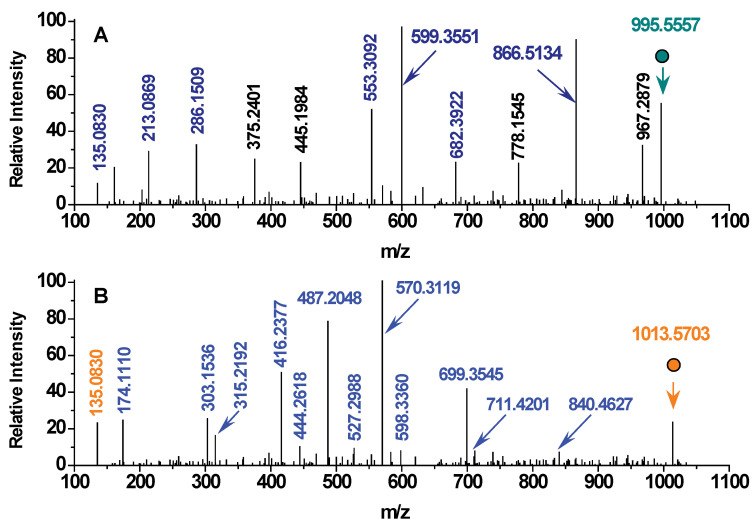
MS/MS spectra for MCLR and MCLR biodegradation products (with linearized MCLR serving as an example). The dots relate to the parent ions MCLR (**A**) and linearized MCLR (**B**).

With the assistance of MS/MS analysis, the MCLR biodegradation products could be further identified according to their secondary structures. Figure 2A shows that the major fragment ions of the single-protonated MCLR (*m*/*z* = 995.5557) were detected at *m*/*z* 135.0809, 213.0869, 286.1509, 397.2081, 470.3125, 553.3092, 599.3551, 682.3922, and 866.5134, corresponding to the secondary structures of [PhCH_2_CH(OCH_3_)]^+^, [(-Glu^6^-Mdha^7^-)+H]^+^, [(-MeAsp^3^-Arg^4^-)+H]^+^, [(-Mdha^7^-Ala^1^-Leu^2^-MeAsp^3^-)+H]^+^/[(-Glu^6^-Mdha^7^-Ala^1^-Leu^2^-)+H]^+^, [(-Arg^4^-Adda^5^-)+H]^+^, [(-Mdha^7^-Ala^1^-Leu^2^-MeAsp^3^-Arg^4^-)+H]^+^, [(-MeAsp^3^-Arg^4^-Adda^5^-)+H]^+^/ [(-Arg^4^-Adda^5^-Glu^6^-)+H]^+^, [(-Arg^4^-Adda^5^-Glu^6^-Mdha^7^-)+H]^+^, and [(-Mdha^7^-Ala^1^-Leu^2^-MeAsp^3^-Arg^4^-Adda^5^-)+H]^+^/[(-Arg^4^-Adda^5^-Glu^6^-Mdha^7^-Ala^1^-Leu^2^-)+H]^+^ [15,16]. For the linearized MCLR with an *m*/*z* = 1013.5703, the detected fragment ions were different from that of MCLR (Figure 2B).

As the linearized MCLR is the direct hydrolysis product of MCLR cleaved at the peptide bond Arg^4^-Adda^5^, -H should be added to the amino terminus of Adda^5^ and –OH should be added to the carboxyl terminus of Arg^4^. Accordingly, the b-type or y-type fragment ions with *m*/*z* at 174.1110, 303.1536, 315.2192, 416.2377, 444.2618, 487.2048, 527.2988, 570.3119, 598.3360, 699.3545, 711.4201, and 840.4627 could be identified as [(-Arg^4^)+H]^+^, [(-MeAsp^3^-Arg^4^)+H]^+^, [(Adda^5^-)+H]^+^, [(-Leu^2^-MeAsp^3^-Arg^4^)+H]^+^, [(Adda^5^-Glu^6^-)+H]^+^, [(-Ala^1^-Leu^2^-MeAsp^3^-Arg^4^)+H]^+^, [(Adda^5^-Glu^6^-Mdha^7^-)+H]^+^, [(-Mdha^7^-Ala^1^-Leu^2^-MeAsp^3^-Arg^4^)+H]^+^, [(Adda^5^-Glu^6^-Mdha^7^-Ala^1^-)+H]^+^, [(-Glu^6^-Mdha^7^-Ala^1^-Leu^2^-MeAsp^3^-Arg^4^)+H]^+^, [(Adda^5^-Glu^6^-Mdha^7^-Ala^1^-Leu^2^-)+H]^+^, and [(Adda^5^-Glu^6^-Mdha^7^-Ala^1^-Leu^2^-MeAsp^3^-)+H]^+^, respectively. Based on the same strategy, other biodegradation products were further identified by comparing their secondary structures with that of the linearized MCLR (see Table 1). For instance, the biodegradation product with an *m*/*z* = 615.3387 not only had the fragment ions [(Adda^5^-Glu^6^-Mdha^7^-)+H]^+^, [(Adda^5^-Glu^6^-)+H]^+^, [(Adda^5^-)+H]^+^, and [PhCH_2_CH(OCH_3_)]^+^, but also had an extra –OH compared to [(Adda^5^-Glu^6^-Mdha^7^-Ala^1^-)+H]^+^ (b-type). Undoubtedly, this biodegradation product was Adda^5^-Glu^6^-Mdha^7^-Ala^1^. It is also worth pointing out that the biodegradation product with an *m*/*z* = 302.1343 could not be identified in this way. In consideration of the theoretical molecular weights for the structural units -Ala-(71.0365 Da), -Glu- (129.0420 Da), and -Mdha- (83.0365 Da), the fragment ions detected at 89.0470, 131.0576, 172.0842, and 214.0947 should be [(-Ala^1^)+H]^+^, [(Glu^6^-)+H]^+^, [(-Mdha^7^-Ala^1^)+H]^+^, and [(Glu^6^-Mdha^7^-)+H]^+^, respectively. Accordingly, the biodegradation product at *m*/*z* = 302.1343 was Glu^6^-Mdha^7^-Ala^1^.

### 2.2. Biological Toxicity Evaluation of the MCLR Biodegradation Products Targeting PP1

To evaluate the detoxification effect of the biodegradation pathway, the MCLR-related biodegradation products in the crude extract were purified with preparative chromatography techniques. The preparation and purification information for the MCLR biodegradation products are listed in Table 2. As the MCLR biodegradation products had a higher purity (>94.2%), they could be directly used for the toxicity evaluation.

**Figure 3:**
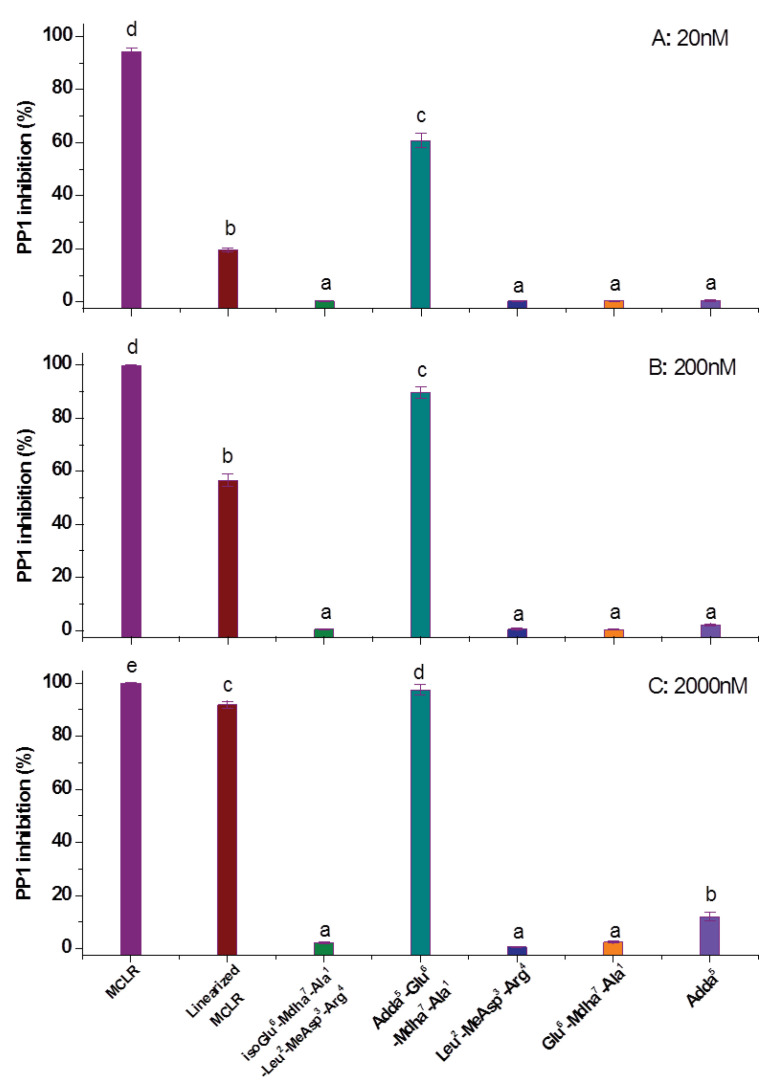
The inhibition effect of the MCLR and MCLR biodegradation products targeting PP1 at different concentrations. The error bar is the standard error of three repeated analyses. Different letters indicate significant differences between groups (*p* < 0.05), obtained using SPSS software.

Based on the PP1 inhibition assay, the inhibition effect for the MCLR and MCLR biodegradation products were obtained (Figure 3). Compared with MCLR (IC_50_ ≈ 1nM), the toxicity of the MCLR biodegradation products decreased in varying degrees. SPSS analysis showed the MCLR and MCLR biodegradation products had significantly different inhibitory effects; there are also differences in toxicity between MCLR and Adda^5^-Glu^6^-Mdha^7^-Ala^1^ (IC_50_ ≈ 12nM), and the linearized MCLR (IC_50_ ≈ 95nM) still had an evident inhibition effect on PP1. Adda^5^ had a certain inhibition effect on PP1 at a higher concentration, while isoGlu^6^-Mdha^7^-Ala^1^-Leu^2^-MeAsp^3^-Arg^4^, isoGlu^6^-Mdha^7^-Ala^1^, and Leu^2^-MeAsp^3^-Arg^4^ had a much lower inhibition effect on PP1. The decreased toxicity of the MCLR biodegradation products showed biodegradation was an effective regulation pathway to control the toxicity of MCLR. However, the potential toxicity of the biodegradation products also deserved further attention.

### 2.3. Molecular Mechanism for the Different Toxicity of MCLR and Its Biodegradation Products Targeting PP1

Although the toxicity experiment revealed biodegradation was an effective pathway, partial MCLR biodegradation products still had an inhibition effect on PP1. The molecular mechanism for the different toxicity of MCLR and the MCLR biodegradation products has not been proposed. With the assistance of molecular docking, the specific interaction between the MCLR/MCLR biodegradation products and PP1 could be further explored.

**Figure 4:**
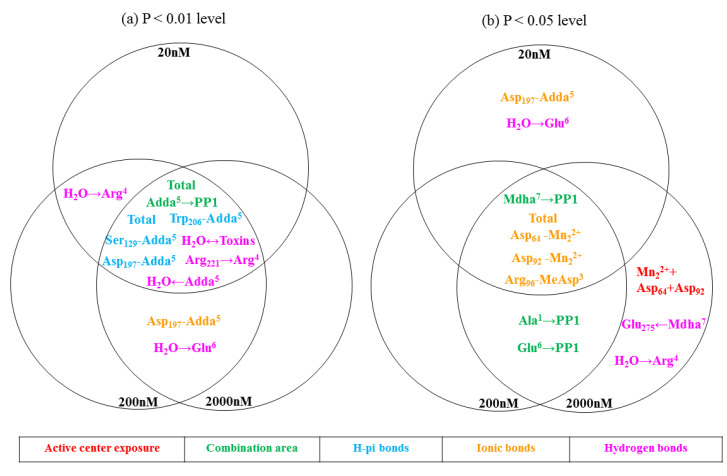
Venn diagrams of the significant parameters at the *p* < 0.01 level (**a**) and *p* < 0.05 level (**b**). Different colors represent different kinds of factors: red for catalytic center exposure, green for binding area, blue for H-pi bonds, orange for ionic bonds, and pink for hydrogen bonds.

The 31 molecular docking parameters for the complexes, including the binding energy, binding areas, exposure area of enzyme catalytic center, hydrogen bonds, ionic bonds, and H-pi bonds, were obtained and listed in Table S1 (see Supporting Information). To assess the regulation mechanism of MCLR biodegradation, the correlation between the molecular docking parameters and toxin toxicity was evaluated by Pearson correlation analysis (regression analysis was not adopted to avoid deleting valid parameters related to a few finite amino-acid residues). As the molecular docking parameters showed diversified correlation with toxin toxicity (see Table 3), the key parameters were confirmed and evaluated by drawing Venn diagrams. Figure 4a (*p* < 0.01) shows that the binding area changes for toxin→PP1, Adda^5^→PP1, the H-pi bonds for PP1↔Adda^5^,Trp_206_↔Adda^5^, Ser_129_↔Adda^5^, and Asp_197_↔Adda^5^, as well as the hydrogen bonds for H_2_O↔Toxins, H_2_O←Adda^5^, and Arg_221_→Arg^4^ were highly and significantly correlated with toxin toxicity at the three test concentrations. The ionic bond for Asp_197_↔Adda^5^ and the hydrogen bond for H_2_O→Glu^6^ were highly and significantly correlated with toxin toxicity at 200 nM and 2000 nM. In turn, the hydrogen bond for H_2_O→Arg^4^ was highly and significantly correlated with toxin toxicity at 20 nM and 200 nM. Figure 4b (*p* < 0.05) shows that the binding area change for Mdha^7^→PP1, the total ionic bonds, and the ionic bonds for Asp_64_ -Mn_2_^2+^, ASP_92_ -Mn_2_^2+^, Arg_96_-MeAsp^3^ were significantly correlated with toxin toxicity at the three test concentrations. The binding area changes for Ala^1^→PP1 and Glu^6^→PP1 were significantly correlated with toxin toxicity at 200 nM and 2000 nM. The ionic bond for Asp_197_↔Adda^5^ and the hydrogen bond for H_2_O→Glu^6^ were significantly correlated with toxin toxicity at 20 nM. The catalytic center exposure area for Mn_2_^2+^+ Asp_64_+Asp_92_ and the hydrogen bonds for H_2_O→Arg^4^ were significantly correlated with toxin toxicity at 2000 nM. Obviously, the above molecular docking parameters (especially the parameters highly correlated with toxin toxicity at the three test concentrations) were important to evaluate the different toxicities of MCLR and the MCLR biodegradation products.

**Figure 5:**
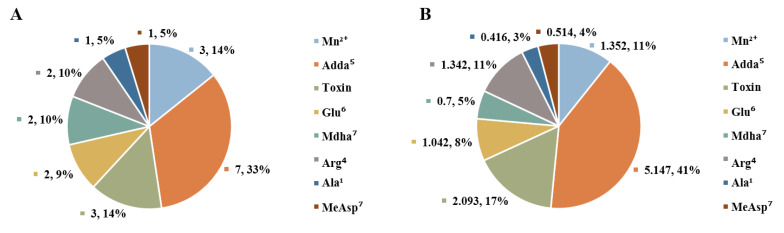
Statistical analysis for the key sites related to the significant parameters: The frequency of the key sites (**A**); The total |R| related to key sites (**B**).

As the toxicity of MCLR and the MCLR biodegradation products were closely related to their “active sites”, the key sites related to the above significant parameters were categorized by pie charts. By counting the frequency of the key sites (Figure 5A), it was found residue Adda^5^ is related to 7 significant parameters (the total H-pi bond should be related to Adda^5^), Mn_2_^2+^ in the catalytic center is related to 3 significant parameters, residues Glu^6^, Mdha^7^, and Arg^4^ are related to 2 significant parameters, and residues Ala^1^ and MeAsp^3^ are related to 1 significant parameter. Besides, the integral toxin is related to 3 significant parameters. By analyzing the Pearson correlation coefficient of the “active site”-related parameters (Figure 5B), a similar rule was found as for the frequency analysis. Parameters related to residue Adda^5^ had a prominent correlation with toxin toxicity; parameters related to the integral toxin, Mn_2_^2+^ ion, and residue Arg^4^ had a large correlation with toxin toxicity; parameters related to residues Glu^6^ and Mdha^7^ had considerable correlation with toxin toxicity; while parameter related to residue Ala^1^ or MeAsp^3^ had a certain correlation with toxin toxicity.

**Figure 6:**
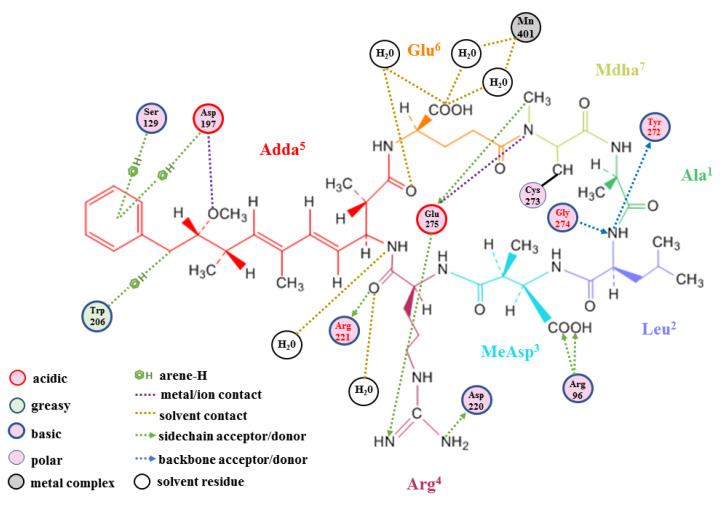
The 2D ligand interaction diagram of the toxins with PP1 (with MCLR-PP1 serving as an example).

Combined with the 2D ligand interaction diagram between the toxins and PP1 (Figure 6), the influence of the active sites was further evaluated. Adda^5^ participated in multiple interactions between the toxins and PP1 and was crucial to the toxicity of MCLR and the MCLR biodegradation products. The evidently reduced toxicity of the “Adda^5^ lost” MCLR biodegradation products fully confirmed this point. The H-pi bonds with Trp_206_, Ser_129_, and Asp_197_, the ionic bond with Asp_197_, and the hydrogen bond with H_2_O promoted the stable binding of Adda^5^ to PP1. Arg^4^, which had an important contribution to the partially significant parameters, bound to PP1 by forming hydrogen bonds with Arg_221_ and H_2_O.

However, the binding area for Arg^4^ targeting PP1 did not have a significant correlation with toxicity (*R* < 0.061, *p* > 0.793). As a consequence, the influence of Arg^4^ on the toxicity of the MCLR biodegradation products was questionable. The following active sites, Glu^6^ and Mdha^7^, bound to PP1 by forming hydrogen bonds with H_2_O and Glu_275_, respectively. As the binding areas of the above sites to PP1 were positively correlated with toxicity, Glu^6^ and Mdha^7^ should have an important influence on the toxicity of MCLR and the MCLR biodegradation products. MeAsp^3^ binding to PP1 merely rely on a single ionic bond with Arg_96_. Besides, there was no significant correlation between toxicity and the binding area of MeAsp^3^ to PP1 (|R| < 0.188, *p* > 0.414). For this reason, the influence of MeAsp^3^ was likely to be marginal. By contrast, Ala^1^ did not have a direct interaction with PP1 but its binding area to PP1 was positively correlated with toxicity. The binding of Ala^1^ to PP1 should be attributed to adjacent active sites. Along with the biodegradation process’s deepening in steps, the interactions between the “lost amino-acid residues” and PP1 could not be obtained. Even so, the total ionic bonds (Asp_197_↔Adda^5^, Arg_96_-MeAsp^3^) and hydrogen bonds (H_2_O→Arg^4^, H_2_O←Adda^5^ and H_2_O→Glu^6^) between the integral toxin and PP1 still showed importance to toxin toxicity. These interactions prompted the binding of MCLR and the MCLR biodegradation products to PP1, and thus exhibit toxic effects. For the two Mn^2+^ ions in the catalytic center, the toxins had an evident influence on the second Mn_2_^2+^ ion (the serial number is defined by the software of PDB). The ionic bonds Asp_64_ -Mn_2_^2+^, ASP_92_ -Mn_2_^2+^, and the catalytic center exposure area for Mn_2_^2+^ + Asp_64_ + Asp_92_ were negatively correlated with toxin toxicity. The introduction of toxins weakened the interaction between Mn_2_^2+^ and Asp_64_/Asp_92_, leading to the inhibition of PP1 catalytic activity. The above key sites and key interactions had important effects on the toxicity of the MCLR/MCLR biodegradation products targeting PP1. In the biodegradation process, the above key sites were lost, and the relevant key interactions weakened or disappeared, resulting in reduced toxicity.

## 3. Conclusions

To explore the regulation effectiveness of the MCLR biodegradation pathway, several typical biodegradation products originated from MCLR were identified, prepared, and purified. Biodegradation was an effective pathway to control the toxicity of MCLR according to the decreased inhibition effect of the MCLR biodegradation products on PP1. However, the secondary toxicity of the partial products (Adda^5^-Glu^6^-Mdha^7^-Ala^1^, linearized MCLR, and Adda^5^) was non-negligible. With the assistance of molecular docking, the specific interactions between the MCLR/MCLR biodegradation products and PP1 were further explored. By analyzing the correlation between the molecular docking parameters and toxin toxicity, it was found that the active sites Adda^5^, Glu^6^, and Mdha^7^ were crucial to the toxicity of MCLR and its biodegradation products. Besides, the ionic bonds and hydrogen bonds between the integral toxin and PP1 also had important effects on the toxin’s toxicity. The bonding of toxins to PP1 also affected the interaction between Mn_2_^2+^ and Asp_64_/Asp_92_, thus exhibiting toxicity. As the biodegradation progresses, the influence of the above key sites and interactions weakened or disappeared, resulting in the reduced toxicity of the biodegradation products in response.

## 4. Materials and Methods

### 4.1. Materials

Acetonitrile, MCLR (98.5%), and trifluoroacetic acid were obtained from Sigma (Saint-Quentin Fallavier, France). PP1 (1200 U/mL) was purchased from EMD Millipore (Darmstadt, Germany). Bovine serum albumin, beef extract peptone medium, MnCl_2_, p-nitrophenyl disodium orthophorphate, tris(hydroxymethyl)aminomethane, and other reagents were purchased from Sinopharm (Shanghai, China).

Toxic *M. aeruginosa* FACHB-905 (producing MCLR) was grown in BG11 medium at 25 °C with a light/dark cycle (12/12). The cultures were harvested at the late exponential phase of growth and had a final cell yield up to about 10^7^ cells/mL [17]. The biodegradation bacterium *Brevibacillus* sp. D1 (GenBank code EU593881), which could effectively remove algae and MCLR, was kindly supplied by Professor Ruimin Mu at Shandong Jianzhu University. *Brevibacillus* sp. D1 was grown in beef extract peptone medium at 35 °C and harvested at OD_420 nM_ ≈ 1.0 (10^9^ cells/mL).

### 4.2. Biodegradation of MCLR

To obtain the biodegradation products of the MCLR, 2000 mL *M. aeruginosa* FACHB-905 was taken and centrifuged at 2000 rpm. The supernatant was filtered through a 1.2 µm GF/C-Whatman glass membrane to remove the cyanobacteria. The filtrate was mixed with 250 mL *Brevibacillus* sp. D1, and incubated at 25 °C for 2–20 days. At regular intervals, a 150 mL biodegradation sample was taken and centrifuged at 2000 rpm. The supernatant was filtered through a 1.2 µm GF/C-Whatman glass membrane to remove the residual biodegradation bacterium. Then the filtrate was divided into several aliquots (about 25 mL/section). *Cleanert* C_18_ solid phase extraction cartridges (500 mg, *Bonna-Agela*) were rinsed with 10 mL acetonitrile and 20 mL water. Each aliquot was added into the conditioned cartridges. The impurities were eluted with 10 mL 10% acetonitrile and the biodegradation products were eluted with 10 mL acetonitrile. Finally, the eluted samples were combined, evaporated to dryness with N_2_ flow, and resuspended with 2 mL acetonitrile. A crude extract of MCLR (not subject to *Brevibacillus* sp. D1) was also prepared as reference.

### 4.3. MCLR Biodegradation Products Analysis

The MCLR biodegradation products in the crude extract were identified with a UHR-TOF mass spectrometer (Bruker Daltonios). The crude extracts were mixed with the same-volume acetonitrile (0.1% trifluoroacetic acid) and were injected into the mass spectrometer with a syringe pump at 3 µL/min. The equipment parameters were set as follows: selected ion scan model, electrospray source voltage 4.4 kV, cone voltage 0.6 kV, desolvation gas N_2_ (0.5 bar), dry gas N_2_ (180 °C, 4.5 L/min), MS acquisition time > 5 s, and MS acquisition accuracy ±10 ppm. By analyzing the secondary ions originating from the MCLR, the MCLR biodegradation products could be further identified by MS/MS. The MS/MS parameters were set as “MS analysis”, except that the full ion scan model (scan range 50–1200) and collision gas N_2_ (collision energy 45–55 eV) were used.

### 4.4. MCLR Biodegradation Products Preparation

The MCLR biodegradation products in the crude extract were further separated using a Dionex Ultimate 3000 HPLC system equipped with an Agilent SB-C_18_ column (9.4 × 250 mm, 5 µm). Firstly, 200 µL of the resuspended sample was injected into the column and eluted by water and acetonitrile (both mobile phases containing 0.1% trifluoroacetic acid). The gradient elution was programmed as follows: 0–5 min, 20% acetonitrile; 35–40 min, 80% acetonitrile; and 40.1–45 min, 20% acetonitrile (35 °C, 2 mL/min). The eluted sample was determined by a UHR-TOF mass spectrometer and the MS parameters were set as in Section 2.3. The separated and purified biodegradation products were collected around specific retention times, evaporated to dryness with N_2_, and dissolved in 100 µL acetonitrile.

### 4.5. Protein Phosphatase 1 Inhibition Assay

The potent biological toxicity of the MCLR biodegradation products was evaluated by a colorimetric protein phosphatase inhibition assay, as modified by Zong et al. [17,18]. Typically, 10 μL PP1 (0.2 U/mL) and 90 μL test samples were mixed in 96-well polystyrene microplate. After 0.5 h, 80 μL p-nitrophenyl disodium orthophorphate (5 mM) was added to the microplate and the samples were incubated for 1 h. The absorbance of the incubated samples was measured with a Thrtmo/max microplate reader. The PP1 activity was calculated by the formula (1 − (A_control_ − A_sample_)/A_control_) × 100%, where A_control_ and A_sample_ were the absorbance of reference sample (without PP1) and test sample at 405 nm, respectively. The experiment was repeated 3 times.

### 4.6. Molecular Docking for the Interaction between Toxins and PP1

Molecular docking for the interaction between the toxins and PP1 was performed with Molecular Operating Environment software (MOE, version 16.09, Shanghai, China). The original models for MCLR-PP1, MCLR, and PP1 were obtained from the Protein Data Bank (PDB code 1FJM, http://www.rcsb.org/pdb/home/home.do). Models for the MCLR biodegradation products were prepared based on the structure of the MCLR. If the PP1 model is defective, the structure of the receptor PP1 needs to be corrected before molecular simulation; PP1 was protonated with hydrogen atoms and ligands (MCLR and its biodegradation products) were introduced and minimized for energy optimization [19]. Then the interactions between the ligands and PP1 were simulated and the experiment condition was set as follows: Amber 10 EHT; Solvation R-Field; reaction temperature 25.0 °C; pH 7.4; and salt 0.05 M. The key parameters, such as binding energies, binding areas, exposure area of the enzyme catalytic center, and the main interaction sites associated with hydrogen-bonds/ionic bonds/H-pi bonds, were obtained to clarify the regulation mechanism of the biodegradation pathway [20].

### 4.7. Statistical Analysis

The correlations between toxin toxicity and the key parameters to ascertain the toxin–PP1 interaction was evaluated by IBM SPSS statistics (version 16.0, Chicago, IL, USA). One-way analysis of variance (ANOVA) was used for significance testing of the data, and an average comparison was made between the different treatment groups. A minimum significance (LSD) test was used.

Pearson correlation analysis was used to analyze the correlation between toxin toxicity and the major molecular docking parameters: (1) Click “Analyze” and expand the drop-down menu; (2) Look for the ”Correlate“ popup menu in the drop-down menu, click ”Bivariate“, and the dialog box “Bivariate Correlations” pops up; (3) Move the source variable on the left to the rectangle under “Variables:” on the right; (4) Check the option “Pearson” in “Correlation Coefficients”. Finally, the correlation results are obtained.

## Figures and Tables

**Table 1:** MS/MS identification of the MCLR biodegradation products.

Detected Ions Identified Products	Ion Types	Linearized MCLR	Glu^6^-Mdha^7^-Ala^1^-Leu^2^-MeAsp^3^-Arg^4^	Adda^5^-Glu^6^-Mdha^7^-Ala^1^	Leu^2^-MeAsp^3^-Arg^4^	Glu^6^-Mdha^7^-Ala^1^	Adda^5^
[M+H]^+^	parent ions	1013.5703	700.3631	615.3387	417.2452	302.1343	332.2218
[PhCH_2_CH(OCH_3_)]^+^	side-chain	135.0809	---	√	---	---	√
[(-Arg^4^)+H]^+^	y-type	174.1110	√	---	√	---	---
[(-MeAsp^3^-Arg^4^)+H]^+^	y-type	303.1536	√	---	√	---	---
[(Adda^5^-)+H]^+^	b-type	315.2192	---	√	---	---	↑+OH
[(-Leu^2^-MeAsp^3^-Arg^4^)+H]^+^	y-type	416.2377	√	---	↑+H	---	---
[(Adda^5^-Glu^6^-)+H]^+^	b-type	444.2618	---	√	---	---	---
[(-Ala^1^-Leu^2^-MeAsp^3^-Arg)^4^+H]^+^	y-type	487.2048	√	---	---	---	---
[(Adda^5^-Glu^6^-Mdha^7^-)+H]^+^	b-type	527.2988	---	√	---	---	---
[(-Mdha^7^-Ala^1^-Leu^2^-MeAsp^3^-Arg^4^)+H]^+^	y-type	570.3119	√	---	---	---	---
[(Adda^5^-Glu^6^-Mdha^7^-Ala^1^-)+H]^+^	b-type	598.3360	---	↑+OH	---	---	---
[(-Glu^6^-Mdha^7^-Ala^1^-Leu^2^-MeAsp^3^-Arg^4^)+H]^+^	y-type	699.3545	↑+H	---	---	---	---
[(Adda^5^-Glu^6^-Mdha^7^-Ala^1^-Leu^2^-)+H]^+^	b-type	711.4201	---	---	---	---	---
[(Adda^5^-Glu^6^-Mdha^7^-Ala^1^-Leu^2^-MeAsp^3^-)+H]+	b-type	840.4627	---	---	---	---	---
[(Glu^6^-)+H]^+^	b-type	---	√	---	---	131.0576	---
[(-Mdha^7^-Ala^1^)+H]^+^	y-type	---	---	√	---	172.0842	---
[(Glu^6^-Mdha^7^-)+H]^+^	b-type	---	√	---	---	214.0947	---
[(-Ala^1^)+H]^+^	y-type	---	---	√	---	89.0470	---

Note: The theoretical molecular weights for the structural units -Ala-, -Leu-, -MeAsp-, -Arg-, -Adda-,-Glu-, and -Mdha- were 71.0365 Da, 113.0835 Da, 129.0420 Da, 156.1005 Da, 313.2036 Da, 129.0420 Da, and 83.0365 Da, respectively. √ means ions with an identical *m*/*z* were detected by the mass spectrograph; --- no related ions with an identical *m*/*z* were detected by the mass spectrograph; ↑ means mass changes were related to these fragment ions.

**Table 2:** Preparation and purification information of the MCLR biodegradation products.

Biodegradation Products	Eluted Times	Biodegradation Times	Total Collection Volumes	Final Concentrations ^a^	Purity ^b^
MCLR (995.5557)	25.26 ± 0.20 min	---	---	---	---
Linearised MCLR (1013.5703)	23.79 ± 0.20 min	4, 6, 8 days	800 × 3 = 2400 µL	41.06 µM/L	94.2%
Glu^6^-Mdha^7^-Ala^1^-Leu^2^-MeAsp^3^-Arg^4^ (700.3631)	20.44 ± 0.20 min	8, 10, 12 days	800 × 3 = 2400 µL	19.71 µM/L	94.8%
Adda^5^-Glu^6^-Mdha^7^-Ala^1^ (615.3387)	19.27 ± 0.20 min	6, 8, 10, 12 days	800 × 4 = 3200 µL	15.93 µM/L	96.0%
Leu^2^-MeAsp^3^-Arg^4^ (417.2452)	12.65 ± 0.20 min	8, 10, 12, 14 days	800 × 4 = 3200 µL	21.10 µM/L	96.7%
Glu^6^-Mdha^7^-Ala^1^ (302.1343)	15.22 ± 0.20 min	12, 14, 16, 18, 20 days	800 × 5 = 4000 µL	7.95 µM/L	94.7%
Adda^5^ (332.2218)	23.18 ± 0.20 min	8, 10, 12, 14, 16, 18, 20 days	800 × 7 = 5600 µL	19.88 µM/L	97.4%

^a^: The final volume for the MCLR biodegradation products was 100 µL; ^b^: sample purity was calculated according to the MS signal intensity of the MCLR biodegradation products and defined as the MCLR biodegradation product/(MCLR+MCLR biodegradation products) × 100%. ---: There is no preparation and purification information.

**Table 3:** Pearson correlation analysis of toxin toxicity and the major molecular docking parameters.

**Pearson Correlation** **Analysis Data ^a^**		**Binding Energy (KJ/Mol)**	**Binding Area (Å^2^)**								**Catalytic Center Exposure (Å^2^)**	
			**Total**	**Ala^1^→PP1**	**Leu^2^→PP1**	**MeAsp^3^→PP1**	**Arg^4^→PP1**	**Adda^5^→PP1**	**Glu^6^→PP1**	**Mdha^7^→PP1**	**Mn_1_^2+^/Mn_2_^2+^**	**Mn_2_^2+^+ Asp_64_+Asp_92_**
Toxicity (20 nM)	R	−0.177	0.746 **	0.364	0.107	−0.158	0.061	0.600 **	0.421	0.455 *	0.001	−0.159
	*p*	0.444	0.000	0.104	0.644	0.494	0.793	0.004	0.057	0.038	0.999	0.492
Toxicity (200 nM)	R	−0.297	0.866 **	0.447 *	0.101	−0.186	0.045	0.712 **	0.485 *	0.519 *	0.001	−0.381
	*p*	0.190	0.000	0.042	0.664	0.420	0.848	0.000	0.026	0.016	0.999	0.088
Toxicity (2000 nM)	R	−0.401	0.919 **	0.437 *	0.125	−0.188	0.060	0.786 **	0.490 *	0.511 *	0.001	−0.531 *
	*p*	0.072	0.000	0.048	0.589	0.414	0.795	0.000	0.024	0.018	0.999	0.109
**Pearson Correlation** **Analysis Data ^a^**		**H-pi bonds (KJ/Mol)**				**Ionic bonds (KJ/Mol)**						
		**Total**	**Trp_206_-Adda^5^**	**Ser_129_-Adda^5^**	**Asp_197_-Adda^5^**	**Total**	**Asp_64_-Mn_2_^2+^**	**Asp_92_-Mn_2_^2+^**	**Arg_96_-MeAsp^3^**	**Asp_220_-Arg^4^**	**Asp_197_-Adda^5^**	**Glu_275_-Mdha^7^**
Toxicity (20 nM)	R	−0.939 **	−0.692 **	−0.884 **	−0.745 **	−0.463 *	−0.455 *	−0.453 *	−0.516 *	−0.161	−0.544 *	−0.100
	*p*	0.000	0.001	0.000	0.000	0.034	0.038	0.039	0.017	0.484	0.011	0.667
Toxicity (200 nM)	R	−0.848 **	−0.786 **	−0.732 **	−0.768 **	−0.498 *	−0.524 *	−0.527 *	−0.534 *	−0.147	−0.695 **	−0.276
	*p*	0.000	0.000	0.000	0.000	0.022	0.015	0.014	0.013	0.526	0.000	0.226
Toxicity (2000 nM)	R	−0.762 **	−0.735 **	−0.615 **	−0.795 **	−0.488 *	−0.510 *	−0.517 *	−0.491 *	−0.166	−0.797 **	−0.374
	*p*	0.000	0.000	0.003	0.000	0.025	0.018	0.016	0.024	0.471	0.000	0.095
**Pearson Correlation** **Analysis Data ^a^**		**Hydrogen bonds (KJ/Mol)**										
		**Total**	**H_2_O↔Toxins**	**H_2_O←Adda^5^**	**H_2_O→Arg^4^**	**H_2_O→Glu^6^**	**Asp_220_←Arg^4^**	**Glu_275_←Arg^4^**	**Glu_275_←Mdha^7^**	**Arg_96_→MeAsp^3^**	**Arg_221_→Arg^4^**	****
Toxicity (20 nM)	R	−0.358	−0.843 **	−0.748 **	−0.807 **	−0.487 *	−0.094	−0.085	0.044	−0.029	−0.799 **	
	*p*	0.111	0.000	0.000	0.000	0.025	0.686	0.737	0.851	0.901	0.000	
Toxicity (200 nM)	R	−0.355	−0.761 **	−0.668 **	−0.624 **	−0.606 **	−0.112	0.023	−0.220	−0.022	−0.669 **	
	*p*	0.114	0.000	0.001	0.003	0.004	0.629	0.929	0.338	0.924	0.002	
Toxicity (2000 nM)	R	−0.306	−0.697 **	−0.597 **	−0.501 *	−0.638 **	−0.156	0.089	−0.440 *	−0.103	−0.627 **	
	*p*	0.109	0.001	0.004	0.021	0.002	0.499	0.725	0.046	0.657	0.005	

^a^: The number of samples is 21 (*n* = 21); R is the Pearson correlation between the molecular simulation parameter and MCLR/MCLR biodegradation products’ toxicity at different toxin levels; *p* is the 2-tailed significance of the related data; ** means significant at the 0.01 level; * means significant at the 0.05 level.

## Supplementary Materials

The following are available online at https://www.mdpi.com/2072-6651/12/12/790/s1, Table S1: Molecular simulation parameters for the complexes of MCLR/MCLR biodegradation products and PP1.
