# Supplementary material for: Regulation Efficacy and Mechanism of the Toxicity of Microcystin-LR Targeting Protein Phosphatase 1 via the Biodegradation Pathway
Source: Toxins (Basel). 2020 Dec 11;12(12):790. doi: 10.3390/toxins12120790 (PMC7764552; doi:10.3390/toxins12120790)
Supplement: Supplementary file 1 [file toxins-12-00790-s001.pdf]

Supplementary Materials: Regulation Efficacy and Mechanism of the Toxicity of Microcystin-LR Targeting Protein Phosphatase 1 via the Biodegradation Pathway

Luyao Ren, Zhengxin Hu, Qian Wang, Yonggang Du and Wansong Zong

Table S1. Molecular simulation parameters for the complexes of MCLR/MCLR biodegradation products and PP1.

Molecular simulation parameters		MCLR	Linearised MCLR	Glu^6-Mdha^7-Ala^1-Leu^2-MeAsp^3-Arg^4	Adda^5-Glu^6-Mdha^7-Ala^1	Leu^2-MeAsp^3-Arg^4	Glu^6-Mdha^7-Ala^1	Adda^5
**Combination energy (KJ/Mol)**		-4820.81	-4882.96	-4801.05	-4765.91	-4837.68	-4684.06	-4764.63
**Combination area (Å^2)**	**Total**	743.5185	752.4809	484.4441	608.3952	369.5579	348.8338	359.8027
	**Ala^1→PP1**	81.8327	89.4847	78.9434	122.8509	---[a]	129.6694	---
	**Leu^2→PP1**	140.4126	145.3441	130.2552	---	153.1681	---	---
	**MeAsp^3→PP1**	66.4691	77.7975	96.1365	---	159.0142	---	---
	**Arg^4→PP1**	129.3682	131.7525	138.4834	---	150.6687	---	---
	**Adda^5→PP1**	351.834	359.5489	---	344.5371	---	---	359.8027
	**Glu^6→PP1**	194.0955	195.9994	193.7585	176.8987	---	196.7631	---
	**Mdha^7→PP1**	137.2425	131.8518	133.6010	142.2166	---	143.4255	---
**Active center exposure (Å^2)**	**Mn_1^{2+}/Mn_2^{2+}**	120.7628	120.7628	120.7628	120.7628	120.7628	120.7628	120.7628

	**Mn_2^{2+}+ Asp_{64}+Asp_{92}**	446.6881	440.0578	449.1638	446.8703	448.3228	445.0868	451.0859
**H-pi bonds (KJ/Mol)**	**Total**	-2.1	-0.5	0	-0.6	0	0	0
	**Trp_{206} - $Adda^5$**	---	---	---	-0.6	---	---	---
	**Ser_{129} - $Adda^5$**	-1.4	---	---	---	---	---	---
	**Asp_{197} - $Adda^5$**	-0.7	-0.5	---	---	---	---	---
**Ionic bonds (KJ/Mol)**	**Total**	-74.4	--69.3	-32.6	-44.3	-69.8	-40.5	-1.5
	**ASP_{64} - Mn_2^{2+}**	-24.5	-24.3	---	-24.1	-24.5	-24.5	
	**ASP_{92} - Mn_2^{2+}**	-11.1	-11.2	---	-11.3	-10.9	-11.2	
	**Arg_{96} - $MeAsp^3$**	-17.2	-13.3	-14.3	-13.5	-15.5	---	---
	**Asp_{220} - Arg^4**	-7.3	-7.0	-6.7	---	-6.6	---	---
	**Asp_{197} - $Adda^5$**	-1.4	-1.8	---	-1.5	---	---	-1.5
	**Glu_{275} - $Mdha^7$**	-4.6	-11.7	-11.6	-7.4	---	-4.8	---
**Hydrogen bonds (KJ/Mol)**	**Total**	-48.5	-43.3	-36.0	-14.0	-37.6	-10.1	-2.2
	**H_2O↔Toxins**	-8.8	-3.8	-2.2	-3.5	---	-3.5	-2.2
	**H_2O←$Adda^5$**	-2.8	---	---	---	---	---	-2.2
	**H_2O→Arg^4**	-2.8	---	---	---	---	---	---
	**H_2O→Glu^6**	-3.2	-3.8	-2.2	-3.5		-3.5	
	**Asp_{220}←Arg^4**	-8.7	-10.2	-8.6	---	-9.1	---	---
	**Glu_{275}←$Mdha^7$**	-0.5	-11.4	---	---	---	-0.5	---
	**Arg_{96}→$MeAsp^3$**	-7.9	-12.2	-10.9	---	-9.8	---	---
	**Arg_{221}→Arg^4**	-11.3	---	---	---	-0.7	---	---

[a] --- no related parameter was detected.
